# A review of data abstraction

**DOI:** 10.3389/frai.2023.1085754

**Published:** 2023-06-23

**Authors:** Gianluca Cima, Marco Console, Maurizio Lenzerini, Antonella Poggi

**Affiliations:** Dipartimento di Ingegneria Informatica, Automatica e Gestionale “A. Ruberti”, Sapienza University of Rome, Rome, Italy

**Keywords:** knowledge representation, abstraction, automated reasoning, data integration, data preparation

## Abstract

It is well-known that Artificial Intelligence (AI), and in particular Machine Learning (ML), is not effective without good data preparation, as also pointed out by the recent wave of data-centric AI. Data preparation is the process of gathering, transforming and cleaning raw data prior to processing and analysis. Since nowadays data often reside in distributed and heterogeneous data sources, the first activity of data preparation requires collecting data from suitable data sources and data services, often distributed and heterogeneous. It is thus essential that providers describe their data services in a way to make them compliant with the FAIR guiding principles, i.e., make them automatically Findable, Accessible, Interoperable, and Reusable (FAIR). The notion of data abstraction has been introduced exactly to meet this need. Abstraction is a kind of reverse engineering task that automatically provides a semantic characterization of a data service made available by a provider. The goal of this paper is to review the results obtained so far in data abstraction, by presenting the formal framework for its definition, reporting about the decidability and complexity of the main theoretical problems concerning abstraction, and discuss open issues and interesting directions for future research.

## 1. Introduction

Despite the increasing centrality of data in AI, the way in which AI deals with data has remained virtually unchanged since the dawn of the discipline. This has to be contrasted with the well-known fact that Artificial Intelligence (AI), and in particular Machine Learning (ML), is not effective without good data preparation, as also pointed out by the recent wave of data-centric AI. The term “data centric” refers to an architecture where data is the primary and permanent asset. So, data preparation precedes the implementation of any given machine learning task, and can potentially support many of such tasks relying on the same domain. More specifically, data preparation is the process of gathering, transforming and cleaning raw data prior to processing and analysis. It is therefore regarded as an important step in any data engineering and data science projects, including machine learning, involving tasks such as understanding, collecting and reformatting data, aggregating, integrating, combining and enriching raw source data and making modifications and corrections in order to meet quality standards.

The first activity of data preparation requires collecting data from suitable data sources and data services, often distributed and heterogeneous. In the era of data as driving asset both for the private and public domain, the availability of services providing data, also called *data services*, is indeed growing incredibly fast. Thus, on one hand, more and more data services are available, on the other hand, more and more AI tasks and applications rely on data services. This scenario opens two crucial issues for data-centric AI. First, from a consumer point of view, how to find the “right” data, i.e., data which properly respond to an information need? Second, from a provider point of view, how to release FAIR-compliant data services, i.e., services automatically Findable, Accessible, Interoperable, and Reusable (FAIR)? An effective answer to the former question is given by exploiting the state of the art technology for *answering queries* over *data integration* systems, which stems from more than thirty years of research. As for the second question, an answer is given by the results on a relatively new service of data integration systems, called *abstraction*. In order to elaborate more on both these answers, let us first make a step back to data integration.

Data integration is the problem of providing a unified and reconciled view of the data stored in a set of autonomous and heterogeneous sources. The theoretical works on data integration systems have advocated a three-layer architecture comprising the *data sources*, which in our setting are the output of the data services, the *global schema*, which is a unified shared conceptualization of the domain of interest, and the *mapping* between the sources and the global schema. Formally, a data integration system is a triple J=〈G,S,M〉, where G is the global schema, S is the source schema and M is the mapping, i.e., a set of logical assertions describing how the data at the sources relate to the elements of the global schema. Then, intuitively, given a set of data sources *D*, J represents all the (possibly incomplete) databases that are instances of G satisfying M w.r.t. *D*.

Once data services have been integrated by means of a data integration system specified through a triple J=〈G,S,M〉, in order to find the “right data,” a data service consumer can rely on query answering. Specifically, by unambiguously expressing an information need as a query over the shared vocabulary of G, he can get the answers that “best correspond” to his need without even having to know the relevant data services. In particular, in most approaches, such answers have been identified as *certain answers*, i.e. answers to $q_{G}$ that would be returned by every database represented by J given a set of data sources *D*. Also, typically, such answers are computed by first reformulating $q_{G}$ in terms of a query $q_{S}$ and then by evaluating $q_{S}$ over *D*. Conversely, in order to make a data service FAIR-compliant, a provider can rely on abstraction over J. Specifically, given a data service originally expressed as a query over a set of data sources, he can get a query over the shared vocabulary of G, that unambiguously describes the data service content, thus making it both accessible, interoperable and reusable. Concretely, given a query $q_{S}$ over the data sources, he would get a query $q_{G}$ over the global schema whose answers “best correspond” to the data service. Obviously, also for abstraction, the meaning of “best correspond” has to be made precise. Ideally, the query $q_{G}$ is the one whose certain answers are exactly the answers of $q_{S}$, for every possible source database. Such a query $q_{G}$ is called perfect *J*-abstraction of $q_{S}$.

We next use an example for informally introducing and illustrating the main notions related to abstraction. In the example, we focus on queries that are conjunctions of atoms, called *conjunctive queries (CQ)*, and unions thereof, called *unions of conjunctive queries (UCQ)*, and we assume that the evaluation of a query expressed over the global schema is based on the certain answer semantics.

** Example 1**. Let J=〈G,S,M〉 be a data integration system where the elements of the source schema S are the predicates (with associated arity) {*s*_1_/1, *s*_2_/2, *s*_3_/1, *s*_4_/1, *s*_5_/2}, the elements of the global schema G are {*g*_1_/2, *g*_2_/1, *g*_3_/2, *g*_4_/2, *g*_5_/1}, and M contains the following assertions (where the free variables are implicitly universally quantified):

$\begin{array}{l} m_{1}:s_{1}(x)\to \exists y.g_{1}(x,y)\\ m_{2}:s_{2}(x,y)\to g_{1}(x,y)\\ m_{3}:s_{3}(x)\wedge s_{4}(x)\to g_{2}(x)\\ m_{4}:s_{3}(x)\wedge s_{2}(x,y)\to g_{3}(x,y)\\ m_{5}:\exists z.s_{5}(y,z)\wedge s_{2}(x,y)\to g_{3}(x,y)\\ m_{6}:s_{5}(x,y)\to g_{4}(x,y)\\ m_{7}:s_{1}(x)\wedge s_{4}(x)\to g_{5}(x) \end{array}$

Consider the query $q_{S}^{1}=\left\{x,y|s_{2}\left(x,y\right)\right\}$. It is easy to see that, for every database *D*, the set of certain answers of $q_{G}^{1}=\left\{x,y|g_{1}\left(x,y\right)\right\}$ coincides with the set of answers of $q_{S}^{1}$ w.r.t. *D*. It follows that the CQ $q_{G}^{1}=\left\{x,y|g_{1}\left(x,y\right)\right\}$ is a perfect J-abstraction of $q_{S}^{1}$.

Consider the query $q_{S}^{2}=\left\{x|\exists y\text{.}s_{2}\left(x,y\right)\right\}$. A natural candidate for the perfect J-abstraction of $q_{S}^{2}$ is $q_{G}^{2}=\left\{x|\exists y\text{.}g_{1}\left(x,y\right)\right\}$. Note, however, that the certain answers to $q_{G}^{2}$ include tuples in *s*_1_ that may not belong to *s*_2_, and therefore $q_{G}^{2}$ is not even a sound J-abstraction of $q_{S}^{2}$ (i.e., it does not retrieve only tuples of $q_{S}^{2}$). Indeed, it can be shown that no UCQ exists that is a perfect J-abstraction of $q_{S}^{2}$. However, the query asking for those *x* such that *g*_1_(*x, y*) *is known to be true, i.e., holds in every model of* J, cannot exploit mapping *m*_1_, and therefore avoids retrieving tuples from *s*_1_. It follows that such query, which is not expressible as a UCQ, is a perfect J-abstraction of $q_{S}^{2}$. Consider the query $q_{S}^{3}=\left\{x|s_{1}\left(x\right)\right\}$. Again, the natural candidate for the perfect J-abstraction of $q_{S}^{3}$ is clearly $q_{G}^{2}=\left\{x|\exists y\text{.}g_{1}\left(x,y\right)\right\}$. However, because of *m*_2_, the certain answers to $q_{G}^{2}$ also include the values in the first component of *s*_2_, and this means that $q_{G}^{2}$ is not a sound J-abstraction of $q_{S}^{3}$, although it is a complete one (i.e., it retrieves all tuples of $q_{S}^{3}$). Another possible candidate is the query $q_{G}^{3}=\left\{x|\exists y\text{.}g_{5}\left(x\right)\right\}$. However, this query captures only the tuples occurring in *s*_1_ which also occur in *s*_4_. It follows that $q_{G}^{3}$ is a sound J-abstraction, although not a complete one. Actually, it can be shown that no perfect J-abstraction of $q_{S}^{3}$ exists in the class UCQ, but $q_{G}^{2}$ and $q_{G}^{3}$ are, respectively, the minimally complete and the maximally sound J-abstraction of $q_{S}^{3}$ in the class UCQ.

Consider now the query $q_{S}^{4}=\left\{\left(\right)|\exists x,y\text{.}s_{5}\left(x,y\right)\wedge s_{3}\left(x\right)\right\}$, and assume that we aim at checking whether its perfect J-abstraction can be expressed as a UCQ. We immediately observe that {()∣∃*x, y*.*g*_4_(*x, y*)∧*g*_2_(*x*)} is a sound J-abstraction of $q_{S}^{4}$. Also, we can easily verify that {()∣∃*x, y, x*_1_.*g*_4_(*x, y*)∧*g*_3_(*x, x*_1_)∧*g*_2_(*x*_1_)} is also sound, and may retrieve tuples that are not retrieved by {()∣∃*x, y*.*g*_4_(*x, y*)∧*g*_2_(*x*)}. More generally, all queries of the form {()∣∃*x, y, x*_1_, …, *x*_*n*_.*g*_4_(*x, y*)∧*g*_3_(*x, x*_1_)∧…∧*g*_3_(*x*_*n*−1_∧*x*_*n*_)∧*g*_2_(*x*_*n*_)}, for *n*≥1, are pairwise incomparable sound J-abstractions of $q_{S}^{4}$. Based on this observation, one can show that there exists no maximally sound J-abstraction of $q_{S}^{4}$ in the class UCQ. However, the following Datalog query (with goal *Ans*) is the maximally sound J-abstraction of $q_{S}^{4}$ in the whole class of monotone queries:


$$\begin{array}{l} \;\;g_{3}(x,y)\;\to \;t_{1}(x,y)\;\;\\ t_{1}(x,y)\wedge t_{1}(y,z)\;\to \;t_{1}(x,z)\;\;\;\Delta \\ g_{4}(x,y)\wedge g_{2}(x)\;\to \;Ans()\;\;\\ g_{4}(x,y)\wedge t_{1}(x,z)\wedge g_{2}(z)\;\to \;Ans() \end{array}$$

We point out that, apart from the scenario of data services providers, data abstraction is relevant in several other contexts. We mention three of them here. In the context of ontology-based data management, abstraction can be used to check whether the mapping provides the right coverage for expressing the relevant data services at the global schema level (Lutz et al., 2018). Also, abstractions can provide the semantics of open datasets and open APIs published by organizations, which is a key aspect for unchaining all the potentials of open data (Cima et al., 2017). Finally, abstraction can be the basis for a semantic-based approach to source profiling (Abedjan et al., 2017), again one of tasks of data preparation, in particular for describing the structure and the content of a data source in terms of the business vocabulary.

The goal of this paper is to review the main notions and results about abstraction. We present the formal framework for its definition, and report about the decidability and complexity of the main theoretical problems concerning abstraction, i.e., verification, existence, and computation. The roadmap of the paper is as follows:

- Section 2 introduces some relevant background about databases, queries, and data integration.
- Section 3 illustrates the formal framework for abstraction in data integration by providing some of the key definitions used throughout the paper.
- Section 4 reports results appearing in Cima et al. (2021) on the relationship between abstraction and another well-studied problem, namely *view-based query processing* (see, e.g., Halevy, 2001). The latter is the problem of answering a query over a schema S in terms of a set of materialized views over S. Interestingly, the established relationship between abstraction and view-based query processing sheds into light new results about both problems.
- Section 5 illustrates results related to the problem of computing best UCQ abstractions of UCQ source queries (Cima et al., 2019). The main results are that, while minimally complete abstractions are guaranteed to exist, this is not the case for maximally sound abstractions. Motivated by the latter result, a restricted scenario is introduced, in which the existence of maximally sound abstractions is always guaranteed.
- Section 6 surveys results on computing best *monotone* abstractions of UCQ source queries (Cima et al., 2022). The principal contributions are the definition of a novel monotone query language (in the context of data integration) and the discussion of how such a language is able to express all forms of the best monotone abstractions (perfect, or approximated).
- Section 7 presents results on computing abstractions of UCQ source queries in a specific, well-known *non-monotone* query language (Cima et al., 2020). The main results are that all forms of best abstractions are not guaranteed to exist in such a language, and, in virtue of this result, two interesting restricted scenarios are investigated.
- Finally, Section 8 concludes the paper by discussing possible future research on abstraction.

## 2. Preliminaries

### 2.1. Databases and queries

We assume a denumerable set of constant symbols *C* that is included in every alphabet that we shall consider. A *database schema* (or simply schema) T is a logical theory, i.e., a finite set of logical axioms, over an alphabet $A_{T}$ of predicate symbols and constants from *C*. A T*-database* is simply a model of T, i.e., an interpretation for $A_{T}$ that satisfies all the axioms of T, with the additional requirements that (*i*) the domain of *D* is *C*, (*ii*) every constant is interpreted into itself, and (*iii*) the extention of every predicate is finite.^1^ In what follows, we will often see a T-database as a finite set of ground facts over $A_{T}$, each of which corresponding to a tuple in the extension of the associated predicate.

As customary, a *database query* over a schema T of arity *n*, or simply an *n*-ary T-query, is a function associating to each T-database a finite set of tuples of constants of arity *n*. Often, however, it is more convenient to specify queries using expressions from some formal language to which a semantics, i.e., an actual query function, is associated. In what follows, whenever we talk about a *query language* L, we mean the class of all queries that can be expressed using L and its associated semantics.

A fundamental query language for our work is the language of *First-Order Logic (FOL) queries*. A FOL query *q* for a schema T is a T-query defined by an expression of the form $\left\{\bar{x}|\phi \left(\bar{x}\right)\right\}$, where $\bar{x}$ is a tuple of variables, called the *distinguished variables* of *q*, and $\phi \left(\bar{x}\right)$ is a FOL formula over alphabet of T containing all the variables in $\bar{x}$. The arity of *q* is the arity of $\bar{x}$, and we will often use $q\left(\bar{x}\right)$ to say that $\bar{x}$ are the free-variables of the FOL query *q* and write $\left\{\bar{x}|\exists ȳ\text{.}\phi \left(\bar{x},ȳ\right)\right\}$ simply as $\phi \left(\bar{x}\right)$. Moreover,we will use the predicate ⊤ to form atoms of any arity; such atoms will always be interpreted as true. Given a T-database *D* and a FOL T-query *q* of arity *n*, *q*^*D*^ is the set of all tuples $\bar{c}\in C^{n}$ such that $D⊧\phi \left(\bar{c}\right)$.

A *conjunctive query (CQ)* *q* over a schema T is a FOL query of the form $\left\{\bar{x}|\exists ȳ\text{.}\phi \left(\bar{x},ȳ\right)\right\}$, where ȳ is a tuple of variables, called the *existential variables* of *q*, and $\phi \left(\bar{x},ȳ\right)$ is a finite conjunction of relational atom. Given a CQ $q=\left\{\bar{x}|\exists ȳ\text{.}\phi \left(\bar{x},ȳ\right)\right\}$, we say that an existential variable *y*∈ȳ is a *join existential variable* of *q* if it occurs more than once in the atoms of $\phi \left(\bar{x},ȳ\right)$. In what follows, we say that a CQ *q* is a *conjunctive query with join-free existential variables (CQJFE)* if there is no join existential variable occurring in *q*.

Other classes of database queries considered in this paper are defined as customary in terms of both syntax and semantics. An *atomic query* is a FOL query where $\phi \left(\bar{x}\right)$ consists of a single relational atom. A *union of conjunctive queries (UCQ)* (resp., *union of conjunctive queries with join-free existential variables (UCQJFE)*) is a query defined as a finite union of CQs (resp., CQJFEs) having the same arity, called its *disjuncts*, and its semantics is defined via the associated FOL query. For the definition of Datalog, Disjunctive Datalog, and Disjunctive Datalog with inequalities (denoted by *DD*^≠^), we refer the reader to Eiter et al. (1997).

### 2.2. Querying sets of databases

In what follows, we will often need to extend the notion of database queries to sets of databases. A *generalized* T*-query* of arity *n* is a function associating to each *set of* T*-databases* a finite set of *n*-tuples of constants in *C*, called the *answers of* *q* *for* Σ and denoted *q*^Σ^. As customary, for two T-queries *q*_1_ and *q*_2_, we write *q*_1_⊑*q*_2_ if $q_{1}^{\sum }\;\subseteq \;q_{2}^{\sum }$ for each set Σ of T-databases, and we write *q*_1_≡*q*_2_ if both *q*_1_⊑*q*_2_ and *q*_2_⊑*q*_1_.

A common method to define a generalized T-query is to lift the semantics of a T-query to sets of T-databases using the notion of *certain answers*. Given a T-query *q* and a set Σ of T-databases, the *certain answers of* *q* *over* Σ are defined as ${⋂}_{D\in \Sigma }q^{D}$. Thus, in what follows, we consider that every generalized T-query is such that given a set Σ of T-databases, *q*^Σ^ is the set of the certain answers of *q* over Σ. This small abuse of notation and the observation that *q*^*D*^ = *q*^{*D*}^ allow us to blur the distinction between queries and generalized queries. Therefore, from now on, unless otherwise specified, we will use the term T-query for generalized T-query.

### 2.3. Data integration

A *data integration system* (Lenzerini, 2002) J is specified by a triple 〈G,S,M〉, where G, the global schema, is a schema over an alphabet $A_{G}$, S, the source schema, is a schema over an alphabet $A_{S}$ (disjoint from $A_{G}$, except for the set *C*), and M is a mapping relating S to G. Specifically, M is a finite set of assertions of the form $q_{S}\to q_{G}$, where $q_{S}$ is an S-query and $q_{G}$ is a G-query of the same arity as $q_{S}$.

The semantics of J is defined relative to an S-database *D*, and, intuitively, is the set of all the G-databases that satisfy M with respect to *D*. A G-database *B* satisfies M with respect to *D*, denoted by (D,B)⊧M, if it satisfies all the assertions in M, i.e., $q_{S}^{D}\subseteq q_{G}^{B}$ for each $\left(q_{S}\to q_{G}\right)\in M$.

Formally, the semantics of J relative to *D*, denoted as mod(J,D), is defined as {B|Bis aG-database such that(D,B)⊧M}. We say that *D* is consistent with J if mod(J,D)≠∅. The answers to a G-query *q* w.r.t. a data integration system J=〈G,S,M〉 and an S-database *D* is simply $q^{mod\left(J,D\right)}$, that we often write simply as $q^{J,D}$. For two G-queries *q*_1_ and *q*_2_, we write $q_{1}{⊑}_{J}\;q_{2}$ if $q_{1}^{J,D}\subseteq q_{2}^{J,D}$ for each S-database *D*; $q_{1}{⊏}_{J}q_{2}$ and J-equivalence are defined accordingly.

Specific classes of mappings considered in the literature are GAV, LAV, GLAV, PGAV, and SPGAV. We introduce them under the assumption that the queries appearing in mapping assertions are conjunctive queries or restricted forms thereof.

A GLAV mapping is a set of assertions of the form $q_{S}\left(\vec{x}\right)\to q_{G}\left(\vec{x}\right)$, where both $q_{S}$ and $q_{G}$ are conjunctive queries over S and G respectively, with distinguished variables $\vec{x}$.

A GAV mapping is a special case of GLAV, constituted by a set of assertions of the form $q_{S}\left(\vec{x}\right)\to A\left(\vec{x}\right)$, where (*i*) $q_{S}$ is a conjunctive query over S and (*ii*) $A\left(\vec{x}\right)$ is an atomic T-query. A pure GAV mapping (PGAV) is a GAV mapping in which each assertion $q_{S}\left(\vec{x}\right)\to A\left(\vec{x}\right)$ is such that no repeated variables appear in $\vec{x}$. A PGAV mapping is called SPGAV (PGAV with single assertion per predicate) if it does not contain a pair of assertions with the same predicate symbol the right-hand side.

A LAV mapping is a special case of GLAV, constituted by a set of assertions $A\left(\vec{x}\right)\to q_{G}\left(\vec{x}\right)$, where (*i*) $A\left(\bar{x}\right)$ is an atomic T-query and (*ii*) $q_{G}$ is a conjunctive query over G with distinguished variables $\vec{x}$.

In what follows, we implicitly refer to a data integration system J=〈G,S,M〉, and when we denote a query by $q_{G}$ (resp., $q_{S}$) we mean that the query is a G-query (resp., S-query).

### 2.4. The EQL-Lite(UCQ) language

*EQL-Lite*(UCQ) is a powerful query language in the context of data integrations systems introduced and studied in Calvanese et al. (2007a). An *EQL-Lite*(UCQ) T-query *q* is an expression of the form $q=\left\{\bar{x}|\varphi \left(\bar{x}\right)\right\}$ where $\varphi \left(\bar{x}\right)$ is an *EQL* formula built according to the following syntax:


$$\varphi (\bar{x})::=\text{K}\varrho |\exists y\text{.}\varphi |\varphi_{1}\wedge \varphi_{2}|\varphi_{1}\vee \varphi_{2}|\neg \varphi$$

with ϱ being a disjunction of conjunction of relational atoms over T possibly involving existentially quantified variables. The semantics is based on the notion of satisfaction of *EQL* sentences w.r.t. *epistemic interpretations*, which are pairs 〈E,I〉 with *E* being a set of interpretations and I∈E. We now inductively define when an epistemic interpretation 〈E,I〉 satisfies an *EQL* sentence φ, written 〈E,I〉 ⊧φ:


〈E,I〉⊨P(c→)        if     I⊨P(c→)〈E,I〉⊨φ1∧φ2   if     〈E,I〉⊨φ1 and 〈E,I〉⊨φ2〈E,I〉⊨¬φ           if     〈E,I〉⊨φ〈E,I〉⊨∃x,φ       if      〈E,I〉⊨φcx for some constant c〈E,I〉⊨Kφ          if      〈E,I′〉⊨φ for every I′∈E,

Then, the answers $q^{J,D}$ of an *EQL-Lite*(UCQ) query $q=\left\{\bar{x}|\varphi \left(\bar{x}\right)\right\}$ w.r.t. a data integration system J=〈G,S,M〉 and an S-database *D* are those tuples $\bar{c}$ of constants such that $\langle \text{mod}\left(J,D\right),B\rangle ⊧\varphi \left(\bar{c}\right)$ for every B∈mod(J,D).

** Example 2**. Consider Example 1 and suppose we are interested in asking for all *x* such that there exists *y* such that we know (*x, y*) belongs to *g*_1_. This can be expressed in *EQL-Lite*(UCQ) as follows:


$$q_{G}^{6}=\{x|\exists y.\text{K}(g_{1}(x,y)\}$$

Note that the query $q_{G}^{6}$ is different from the query asking for all *x* such that we know there exists *y* such that (*x, y*) belongs to *g*_1_, which is expressed as follows:


$$q_{G}^{7}=\{x|\text{K}(\exists y\text{.}g_{1}(x,y)\}$$

Indeed, while it can be verified that the answers to $q_{G}^{6}$ over J coincide with the answers to the query $q_{S}^{2}=\left\{x|\exists y\text{.}s_{2}\left(x,y\right)\right\}$, the answers to $q_{G}^{7}$ over J coincide with the answers to the query $q_{S}^{5}=q_{S}^{2}\cup \left\{x|s_{1}\left(x\right)\right\}$.

## 3. Framework

We proceed to introduce the notion of query abstraction following Cima et al. (2019) for the basic definitions. We say that $q_{G}$ is a *perfect* J*-abstraction of* $q_{S}$ if $q_{G}^{J,D}=q_{S}^{D}$, for each S-database *D* consistent with J. Clearly, if a perfect J-abstraction of $q_{S}$ exists, then it is unique up to J-equivalence, i.e., if *q*′ is a perfect J-abstraction of $q_{S}$ then $q^{\prime }{=}_{J}q_{G}$. Therefore in the following we will talk about *the* perfect J-abstraction of $q_{S}$.

** Example 3**. Consider Example 1. It is easy to verify that $q_{G}^{1}$ is the perfect J-abstraction of $q_{S}^{1}$.

The following theorem presents a preliminary characterization of the existence of perfect J-abstractions.

** Theorem 1**. [(Cima et al., 2021, Theorem 1)] There exists a perfect J-abstraction of $q_{S}$ if and only if for all pair *D, D*′ of S-databases, $mod\left(J,D\right)=mod\left(J,D^{\prime }\right)$ implies $q_{S}^{D}=q_{S}^{D^{\prime }}$.

As the condition of being a perfect J-abstraction of source query is rather strong one, it might be very well the case that such a global schema query may not exist.

** Example 4**. Consider again Example 1. Using Theorem 1, we can show that there exists no perfect J-abstraction for $q_{S}^{4}$. In fact, for the databases *D* = {*s*_5_(*a, b*)} and $D^{\prime }=\left\{s_{5}\left(a,b\right),s_{3}\left(a\right)\right\}$, we have $mod\left(J,D\right)=mod\left(J,D^{\prime }\right)$ but $D⊭q_{S}^{5}$ while $D^{\prime }⊧q_{S}^{5}$.

In these cases, it is reasonable to consider weaker notions, such as sound or complete approximations of perfectness. We say that $q_{G}$ is a *complete* J*-abstraction of* $q_{S}$ if $q_{S}^{D}\subseteq q_{G}^{J,D}$, for each S-database *D* consistent with J. Similarly, we say that $q_{G}$ is a *sound* J*-abstraction of* $q_{S}$ if $q_{G}^{J,D}\subseteq q_{S}^{D}$, for each S-database *D* consistent with J. Obviously, one is interested in complete or sound abstractions that approximate $q_{S}$ *at best*, at least in the context of a specific class of queries. If $L_{G}$ is a class of queries, we say that a global schema query $q_{G}\in L_{G}$ is an $L_{G}$*-minimally complete* J*-abstraction of* $q_{S}$ if $q_{G}$ is a complete J-abstraction of $q_{S}$ and there is no global schema query $q_{G}^{\prime }\in L_{G}$ such that $q_{G}^{\prime }$ is a complete J-abstraction of $q_{S}$ and $q_{G}^{\prime }{⊏}_{J}q_{G}$. Similarly, we say that a global schema query $q_{G}\in L_{G}$ is an $L_{G}$*-maximally sound* J*-abstraction of* $q_{S}$ if $q_{G}$ is a sound J-abstraction of $q_{S}$ and there is no global schema query $q_{G}^{\prime }\in L_{G}$ such that $q_{G}^{\prime }$ is a sound J-abstraction of $q_{S}$ and resp., $q_{G}{⊏}_{J}q_{G}^{\prime }$.

** Example 5**. Consider again Example 1. Queries $q_{G}^{2}$ and $q_{G}^{3}$ are, respectively, the UCQ-minimally complete and UCQ-maximally sound J-abstraction of $q_{S}^{3}$.

Depending on the chosen language $L_{G}$, it may be the case that no $L_{G}$-minimally complete or $L_{G}$-maximally sound J-abstraction exists (see again Example 1 for some concrete cases). Moreover, even if one such abstraction exists, it may not be unique. For some classes Q of queries, however, one can show that a Q-maximally sound (resp., Q-minimally complete) J-abstraction of $q_{S}$ exists, then it is unique up to J-equivalence. This is the case, for example, of the class of UCQs for which, if a UCQ-maximally sound (resp., UCQ-minimally complete) J-abstraction of exists, then it is unique up to J-equivalence. Thus, in the following, we simply talk about *the* UCQ-maximally sound and *the* UCQ-minimally complete J-abstraction of a source query $q_{S}$. Other classes of queries with this properties will be introduced in the subsequent sections.

In the next sections, we will study J-abstraction for data integration systems of a specific form, namely where (*i*) the mapping is of type GLAV or special cases of GLAV, and (*ii*) if not otherwise stated, the set of axioms of both the global schema and the source schema is empty. Also, we will limit our analysis to abstractions of UCQ source queries.

## 4. View-based query processing and query abstraction

It is well-known that there is a relationship between data integration and view-based query processing, grounded on the idea that the sources of a LAV data integration systems can be considered as views defined over the global schema, in particular sound views (Lenzerini, 2002). In this section, we take another approach and establish a relationship between GAV data integration systems and views, based on the idea that the elements of the global schema can be considered as views defined over the source schema.

This section is organized as follows. We first recall the basic notions about view-based query processing. Then, in Section 4.1 we make clear the relationship between GAV data integration systems and views, while in Section 4.2 we establish the connection between abstractions and rewriting queries using views. Finally, in Sections 4.3 and 4.4 we use the above connection to introduce results for abstraction and view-based query processing, respectively. All the results presented in this section appear in Cima et al. (2021).

View-based query processing is a general term denoting several tasks related to the presence of views in databases. A set of views V over a schema T is constituted by a finite set of view predicate symbols, where each V∈V has a specific arity, and an associated *view definition* $V_{T}$, i.e., a query over T of the same arity of *V*. An extension of a view *V* is simply a set of facts for *V*, and a V*-extension* E is constituted by an extension for each view in V. Given a T-database *D*, we denote by V(D) the V-extension $\left\{V\left(\bar{c}\right)|V\in V\text{and}\bar{c}\in V_{T}^{D}\right\}$. In what follows, we use the term L views to indicate a set of views in which all view definitions are queries expressed in the query language L.

Two particular notions have been subject to extensive investigations in the view-based processing literature, namely *view-based query rewriting* and *view-based query answering* (Calvanese et al., 2000, 2007b).

In the former notion, originated in Levy et al. (1995), we are given a query $q_{T}$ over a schema T and a set of views V over T, and the goal is to reformulate $q_{T}$ into a query $q_{V}$, called a V-rewriting, in terms of the view predicate symbols of V. We obtain different variants of V-rewritings depending on the relationship between $q_{T}$ and $q_{V}$ we aim at. We call $q_{V}$ (*i*) a V*-rewriting of* $q_{T}$ *under exact views*, or simply V*-rewriting of* $q_{T}$, if for every T-database *D* it holds that $q_{V}^{V\left(D\right)}\subseteq q_{T}^{D}$, (*ii*) an *exact* V*-rewriting of* $q_{T}$ if for every T-database *D* it holds that $q_{V}^{V\left(D\right)}=q_{T}^{D}$. Note that, if we fix a specific query language $L_{V}$ for expressing V-rewritings, we might lose power in expressing V-rewritings. In this case, a reasonable goal is to compute V-rewritings expressible in $L_{V}$ that are “maximal” in the class $L_{V}$. Formally, we say that a query $q_{V}\in L_{V}$ is an $L_{V}$*-maximal* V*-rewriting of* $q_{T}$, if (*i*) $q_{V}$ is a V-rewriting of $q_{T}$; and (*ii*) there is no $q_{1}\in L_{V}$ such that (*a*) *q*_1_ is a V-rewriting of $q_{T}$, (*b*) $q_{V}^{V\left(D\right)}\subseteq q_{1}^{V\left(D\right)}$ for each T-database *D*, and (*c*) there is a T-database *D* for which $q_{V}^{V\left(D\right)}⊊q_{1}^{V\left(D\right)}$.

As argued in Nash et al. (2010), given $q_{T}$ and V, the problem of checking whether there exists an exact V-rewriting of $q_{T}$ (called *losslessness with respect to rewriting* Calvanese et al., 2007b) is equivalent to the problem, called *view determinacy* (Nash et al., 2010), of checking whether $q_{T}$ is determined by V, denoted $V↠q_{T}$, i.e., whether $V\left(D_{1}\right)=V\left(D_{2}\right)$ implies $q_{T}^{D_{1}}=q_{T}^{D_{2}}$ for each pair of T-databases *D*_1_ and *D*_2_. Indeed, on the one hand, if $V↠q_{T}$, then the function $q_{V}$ associating to each V(D) the tuples $q_{T}^{D}$, for each T-database *D*, is an exact V-rewriting of $q_{T}$, on the other hand, if $V↠̸q_{T}$, then such $q_{V}$ is not a function, and hence an exact V-rewriting of $q_{S}$ cannot exist.

In the view-based query answering, originated in Duschka and Genesereth (1997), besides $q_{T}$ and V we are also given a V-extension E, and the goal is to compute the so-called *certain answers of* $q_{T}$ *w.r.t*. V *and* E, denoted by ${\text{cert}}_{q_{T},V}^{E}$, which are those tuples of constants $\bar{c}$ such that $\bar{c}\in q_{T}^{D}$ for each S-database *D* satisfying E⊆V(D). We denote by ${\text{cert}}_{q_{T},V}$ the query over V that, for every V-extension E, computes the certain answers of $q_{T}$ w.r.t. V and E, and we call ${\text{cert}}_{q_{T},V}$ the *perfect* V*-rewriting of* $q_{T}$ *under sound views*, or simply *perfect* V*-rewriting of* $q_{T}$.

### 4.1. View-based query processing and data integration

We start by describing how to obtain, from any data integration system J with PGAV mapping, a suitable set of UCQ views^2^ $V_{J}$, and, viceversa, from any set of UCQ views V, a suitable data integration system $J_{V}$ with PGAV mapping.

For a data integration system J=〈G,S,M〉 with M∈PGAV, the set of UCQ views $V_{J}$ is such that (*i*) the set of view symbols coincides with $A_{G}$, and (*ii*) for each view symbol *g*, the associated view definition $g_{S}$ is the following UCQ over S:


$$\left\{\bar{x_{1}}|\exists \bar{y_{1}}\text{.}\phi_{S}^{1}\left(\bar{x_{1}},\bar{y_{1}}\right)\right\}\cup \ldots \cup \left\{\bar{x_{l}}|\exists \bar{y_{l}}\text{.}\phi_{S}^{l}\left(\bar{x_{l}},\bar{y_{l}}\right)\right\},$$

where we have one disjunct $\exists \bar{y_{i}}\text{.}\phi_{S}^{i}\left(\bar{x_{i}},\bar{y_{i}}\right)$ for each mapping assertion in M of the form $\exists \bar{y_{i}}\text{.}\phi_{S}^{i}\left(\bar{x_{i}},\bar{y_{i}}\right)\to g\left(\bar{x_{i}}\right)$. Note that, if M∈SPGAV, then all view definitions in $V_{J}$ are CQs.

** Example 6**. Let J=〈G,S,M〉 be a data integration system such that $M=\left\{m_{1},m_{2},m_{3}\right\}$ with:


$$\begin{array}{l} m_{1}:\exists y_{1},y_{2}\text{.}s_{1}\left(y_{1},x,x\right)\wedge s_{2}\left(x,y_{2},y_{2}\right)\to g_{1}\left(x\right)\\ m_{2}:\exists y_{1},y_{2},y_{3}\text{.}s_{1}\left(y_{1},x_{1},x_{2}\right)\wedge s_{2}\left(x_{2},y_{2},y_{3}\right)\to g_{2}\left(x_{1},x_{2}\right)\\ m_{3}:\exists y_{1}\text{.}s_{3}\left(x_{1},x_{2},y_{1}\right)\to g_{2}\left(x_{1},x_{2}\right) \end{array}$$

Then, the UCQ views $V_{J}$ over S is $V_{J}=\left\{g_{1},g_{2}\right\}$, where ${g_{1}}_{S}=\left\{x|\exists y_{1},y_{2}\text{.}s_{1}\left(y_{1},x,x\right)\wedge s_{2}\left(x,y_{2},y_{2}\right)\right\}$ and ${g_{2}}_{S}=\left\{x_{1},x_{2}|\exists y_{1},y_{2},y_{3}\text{.}s_{1}\left(y_{1},x_{1},x_{2}\right)\wedge s_{2}\left(x_{2},y_{2},y_{3}\right)\right\}\cup \left\{x_{1},x_{2}|\exists y_{1}\text{.}s_{3}\left(x_{1},x_{2},y_{1}\right)\right\}$.

For a set of UCQ views V over a schema S, the data integration system $J_{V}=\langle G,S,M\rangle$ is such that (*i*) $A_{G}$ coincides with the view predicate symbols in V, (*ii*) G has no axiom, and (*iii*) M is defined as follows: for each view symbol V∈V and for each CQ $\left\{\bar{x}|\exists ȳ\text{.}\phi_{S}\left(\bar{x},ȳ\right)\right\}$ that is a disjunct in the UCQ $V_{S}$, the mapping M includes a mapping assertion of the form: $\exists ȳ\text{.}\phi_{S}\left(\bar{x},ȳ\right)\to V\left(\bar{x}\right).$ Note that, in general, M∈PGAV. However, if V is a set of CQ views, then M∈SPGAV.

** Example 7**. Let $V=\left\{V_{1},V_{2}\right\}$ be a set of UCQ views over S such that: ${V_{1}}_{S}=\left\{x_{1},x_{2},x_{3}|s_{3}\left(x_{1},x_{2},x_{3}\right)\right\}\;\cup \;\left\{x_{1},x_{2},x_{3}|\exists y\text{.}s_{1}\left(x_{1},y\right)\wedge s_{2}\left(y,x_{2},x_{3}\right)\right\}\;\cup \;\left\{x_{1},x_{2},x_{3}|\exists y_{1},y_{2}\text{.}s_{1}\left(x_{1},y_{1}\right)\wedge s_{4}\left(y_{2},x_{2},x_{3}\right)\right\}$ and ${V_{2}}_{S}=\left\{x_{1},x_{2},x_{3},x_{4}|\exists y\text{.}s_{1}\left(x_{1},x_{2},y\right)\wedge s_{3}\left(y,x_{3},x_{4}\right)\right\}$.

Then, the data integration system is $J_{V}=\langle G,S,M\rangle$, where $A_{G}=\left\{V_{1},V_{2}\right\}$ and $M=\left\{m_{1},m_{2},m_{3},m_{4}\right\}$ with:


$$\begin{array}{l} m_{1}:s_{3}\left(x_{1},x_{2},x_{3}\right)\to V_{1}\left(x_{1},x_{2},x_{3}\right),\\ m_{2}:\exists y\text{.}s_{1}\left(x_{1},y\right)\wedge s_{2}\left(y,x_{2},x_{3}\right)\to V_{1}\left(x_{1},x_{2},x_{3}\right),\\ m_{3}:\exists y_{1},y_{2}\text{.}s_{1}\left(x_{1},y_{1}\right)\wedge s_{4}\left(y_{2},x_{2},x_{3}\right)\to V_{1}\left(x_{1},x_{2},x_{3}\right),\\ m_{4}:\exists y\text{.}s_{2}\left(x_{1},x_{2},y\right)\wedge s_{4}\left(y,x_{3},x_{4}\right)\to V_{2}\left(x_{1},x_{2},x_{3},x_{4}\right). \end{array}$$

For a data integration system J with PGAV mapping and a set of UCQ views V, the pair (J,V) is said to be *coherent* if (*i*) the schema over which the set of views V is defined and the source of J coincide, and (*ii*) $J=J_{V}$ or $V=V_{J}$. In what follows, when we talk about a coherent pair (J,V), we use S to denote the common schema between J and V.

Based on the relationship between $J_{V}$ and $V_{J}$, the following proposition provides a connection between existence of perfect abstractions and existence of exact rewritings.

** Proposition 1**. [(Cima et al., 2021, Proposition 1)] If (J,V) is a coherent pair and $q_{S}$ is an S-query, then there exists a perfect J-abstraction of $q_{S}$ if and only if there exists an exact V-rewriting of $q_{S}$.

### 4.2. Abstractions and rewritings of *DD*^≠^

We now turn our attention to a concrete class of queries, namely *DD*^≠^. From now on, when we use L, we refer to a sublanguage of *DD*^≠^. By exploiting well-known results, we provide connections between the notion of J-abstractions and V-rewritings in the context of *DD*^≠^ and its sublanguages. To this end, we first introduce some terminology.

Given a mapping ℳ∈PGAV relating S to G and a G-query *q* in a certain query language L, the M*-unfolding of* *q* (Lenzerini, 2002), denoted by ${\text{unf}}_{M}\left(\text{q}\right)$, is the S-query obtained by replacing each atom α occurring in the expression corresponding to *q* by the logical disjunction of all the left-hand sides of the mapping assertions in M having the predicate symbol of α in the right-hand side (being careful to use unique variables in place of those variables that appear in the left-hand side of the mapping assertions but not in the right-hand side of those).

Given a set of UCQ views V over S and a V-query *q* in a certain query language L, the V*-expansion of* *q* (Levy et al., 1995), denoted by ${\text{exp}}_{V}\left(\text{q}\right)$, is the S-query obtained by replacing each atom α occurring in in the expression corresponding to *q* by the view definition associated to the view predicate name of α (again, being careful to use unique variables in place of those variables that appear in the bodies of the view but not in the heads of those).

** Proposition 2**. [(Cima et al., 2021, Proposition 2)] If (J,V) is a coherent pair, $q_{S}$ is an S-query in L, and *q* is a query in L, then *q* is a sound (resp., perfect) J-abstraction of $q_{S}$ if and only if *q* is a V-rewriting (resp.,exact V-rewriting) of $q_{S}$.

Actually, as shown in Duschka and Genesereth (1998, Lemma 1), if L allows for the union operator, then for any pair of UCQ views V over S and query $q_{S}\in L$ over S, if an L-maximal V-rewriting of $q_{S}$ exists, then it is unique up to V-equivalence, and, moreover, it coincides with the perfect V-rewriting of $q_{S}$.^3^ From Proposition 2 and the above observation, we can derive the following result.

** Corollary 1**. [(Cima et al., 2021, Corollary 1)] If (J,V) is a coherent pair and L allows for the union operator, then for every pair of queries $q_{S},q\in L$, we have that *q* is the L-maximally sound J-abstraction of $q_{S}$ if and only if *q* is the perfect V-rewriting of $q_{S}$.

By exploiting the above provided relationships, we are now ready to investigate how results and techniques from the view-based query processing literature can be directly translated into results and techniques in the context of abstraction, and viceversa.

### 4.3. From view-based query processing to abstraction

By combining Proposition 1 with a well-known undecidability result about view determinacy, we can derive a negative result about an arguably fundamental problem for the notion of abstraction, namely the *existence problem* (with no restrictions on the query language to express perfect abstractions) of perfect abstractions, even in very restricted settings.

** Theorem 2**. [(Cima et al., 2021, Theorem 2)] Given a data integration system J=〈G,S,M〉 with M∈SPGAV and a CQ S-query $q_{S}$, checking whether there exists a perfect J-abstraction of $q_{S}$ is undecidable.

By exploiting Corollary 1, we now illustrate how to use off-the-shelf algorithms for rewriting queries in the presence of views as algorithms for computing abstractions. By results of Levy et al. (1995), for CQ views V, perfect V-rewritings of UCQs $q_{S}$ can be always expressed as UCQs, and can be always computed [e.g., by means of the *bucket* algorithm (Levy et al., 1996) or the *MiniCon* algorithm (Pottinger and Halevy, 2001)]. Thus Corollary 1 implies that, given a data integration system J=〈G,S,M〉 with M∈SPGAV and a UCQ S-query $q_{S}$, we can compute the UCQ-maximally sound J-abstraction of $q_{S}$ as follows: (*i*) compute $V_{J}$, and (*ii*) compute and return the UCQ corresponding to the perfect $V_{J}$-rewriting of $q_{S}$.

** Corollary 2**. [(Cima et al., 2021, Corollary 2)] If J is a data integration system with SPGAV mapping and $q_{S}$ is a UCQ S-query, then the UCQ-maximally sound J-abstraction of $q_{S}$ exists and is computable.

Things get more complicated when we consider a data integration system J with PGAV mappings, which are clearly more expressive than SPGAV, for which $V_{J}$ is a set of UCQ views, rather than CQ views. Indeed, for UCQ views V, UCQ-maximal V-rewritings of CQs $q_{S}$ are not guaranteed to exist (Duschka and Genesereth, 1998; Afrati and Chirkova, 2019), and thus, in general, perfect V-rewritings of CQs $q_{S}$ are not expressible as UCQs. However, the perfect V-rewritings of UCQs (actually, even of *Datalog* queries) $q_{S}$ can always be expressed in *DD*^≠^, and can always be computed using the technique presented in Duschka and Genesereth (1998). Thus, Corollary 1 implies that, given a data integration system J=〈G,S,M〉 with M∈PGAV and a UCQ S-query $q_{S}$, we can compute the *DD*^≠^-maximally sound J-abstraction of $q_{S}$ as follows: (*i*) compute $V_{J}$, and (*ii*) compute and return the *DD*^≠^ query corresponding to the perfect $V_{J}$-rewriting of $q_{S}$.

** Corollary 3**. [(Cima et al., 2021, Corollary 3)] If J is a data integration system with PGAV mapping and $q_{S}$ is a UCQ S-query, then the *DD*^≠^-maximally sound J-abstraction of $q_{S}$ exists and is computable.

### 4.4. From abstraction to view-based query processing

As already observed, Duschka and Genesereth (1998) and Afrati and Chirkova (2019) show that for a given set V of UCQ views, UCQ-maximal V-rewritings of CQs may not exist. Combined with an observation made above, this means that perfect V-rewritings of CQs are in general not expressible as UCQs. We point out that the CQ $q_{S}$ used to prove such results contain more than one join existential variable. As a consequence, in the case of UCQ views V, it is still open whether (*i*) the result holds even for $q_{S}$ with just one join existential variable (*ii*) perfect V-rewritings of UCQJFEs are expressible as UCQs. By combining Corollary 1 with results of Cima et al. (2019) (that we will discuss in Section 5), we can actually answer positively to both questions.

** Corollary 4**. [(Cima et al., 2021, Corollary 4)] For a set V of UCQ views, the UCQ-maximal V-rewritings of $q_{S}$ may not exist, even if $q_{S}$ is a CQ with one join existential variable.

On the other hand, in Section 5, we will show that for a data integration systems J with PGAV mapping, UCQ-maximally sound J-abstractions of UCQJFEs are guaranteed to exist, and we will provide an algorithm to compute them (Theorem 5). Thus, given a set of UCQ views V over a schema S and a UCQJFE S-query $q_{S}$, we can compute the perfect V-rewriting of $q_{S}$ as follows: (*i*) compute $J_{V}$, and (*ii*) compute and return the UCQ-maximally sound $J_{V}$-abstraction of $q_{S}$. This leads to the following positive result for V-rewritings of UCQJFEs.

** Corollary 5**. [(Cima et al., 2021, Corollary 5)] If V is a set of UCQ views and $q_{S}$ is a UCQJFE S-query, then the perfect V-rewriting of $q_{S}$ is computable and can be expressed as a UCQ.

## 5. UCQ abstractions

In this section we investigate the problem of checking the existence of abstractions in the class UCQ, and of their computation. We first study the case of UCQ-minimally complete J-abstractions, then we switch to UCQ-maximally sound J-abstractions, and finally we tackle perfect J-abstractions in the class UCQ. We observe that all the results presented in this section appear in Cima et al. (2019).

On the positive side, we show that UCQ-minimally complete abstractions always exist, by providing an algorithm to compute them. In a nutshell, given a data integration system J=〈G,S,M〉 and a UCQ $q_{S}=q_{S}^{1}\cup \ldots \cup q_{S}^{n}$, an algorithm to compute the UCQ minimally-complete J-abstraction of $q_{S}$ returns the union of CQs of the form $\left\{\bar{x_{i}}|\exists \bar{Y_{i}}\text{.}M\left(q_{S}^{i}\right)\wedge ⊤\left(\bar{x_{i}}\right)\right\}$ obtained by simply “applying” the mapping M to each CQ $q_{S}^{i}$ in $q_{S}$, using ⊤ to bind the distinguished variables that are not involved in the application of M to $q_{S}^{i}$. Formally, applying the GLAV mapping M to a CQ *q* means to chase (Fagin et al., 2005) the atoms in *q* by using the tuple generating dependencies corresponding to the assertions in M.

** Theorem 3**. [(Cima et al., 2019, Theorem 13)] The UCQ-minimally complete J-abstraction of $q_{S}$ always exists and is computable.

On the negative side, the following shows that UCQ-maximally sound abstractions may not exist.

** Theorem 4**. [(Cima et al., 2019, Theorem 16)] The UCQ-maximally sound J-abstractions of $q_{S}$ may not exist if at least one of the following is true:

(a) $q_{S}$ contains a join existential variable;
(b) M contains a LAV mapping assertion;
(c) M contains a non-PGAV mapping assertion.

Interestingly, in order to illustrate the case (a) of the above theorem we can refer to a slight modification of the data integration system J introduced in Example 1. In particular, let $J_{1}=\langle G,S,M_{1}\rangle$ be obtained from J by removing from M the mapping *m*_1_, and consider the query $q_{S}^{4}$ of Example 1. Note that $M_{1}\in PGAV$ and $q_{S}^{4}$ contains a join existential variable, *x*. Clearly, removing *m*_1_ has no impact on the abstraction of $q_{S}^{4}$. Thus, as already discussed in Example 1, there exists no UCQ-maximally sound $J_{1}$-abstraction of $q_{S}^{4}$.

Motivated by Theorem 4, we next introduce a specific scenario, that we call *restricted*, obtained from the general one by limiting the mapping language to PGAV, and $q_{S}$ to be UCQJFEs. It can be shown that for such a restricted scenario, UCQ-maximally sound abstractions always exist. Intuitively, the latter can be derived by showing that for any UCQJFE $q_{S}$ and data integration system J=〈G,S,M〉 with M∈PGAV, a CQ-maximally sound J-abstraction of $q_{S}$ may comprise at most $k_{q_{S}}^{M}$ atoms, where $k_{q_{S}}^{M}$ is an integer that depends on the number of atoms occurring in $q_{S}$ and the number of mapping assertions occurring in M. Hence, given a data integration system J with PGAV mapping and an UCQJFE $q_{S}$, an algorithm to compute the UCQ-maximally sound J-abstraction of $q_{S}$ simply returns the union of all CQs $q_{G}$ comprising at most $k_{q_{S}}^{M}$ atoms, that are sound J-abstractions of $q_{S}$. The crucial observation here is that in order to check whether $q_{G}$ is a sound J-abstraction of $q_{S}$, it is sufficient to check whether $unf_{ℳ}(q_{G})$ $⊑q_{S}$, which is decidable, since both $q_{S}$ and $unf_{ℳ}(q_{G})$ are UCQs (Sagiv and Yannakakis, 1980).

** Theorem 5**. [(Cima et al., 2019, Theorem 21)] In the restricted scenario, the UCQ-maximally sound J-abstractions of $q_{S}$ always exists and is computable.

To conclude the section, we provide the last positive result about perfect abstractions in the class UCQ. Namely, we show that checking whether there exists a UCQ that is the perfect J-abstraction of $q_{S}$ is decidable. In particular, given a data integration system J with GLAV mapping and a UCQ $q_{S}$, an algorithm to decide whether there exists a UCQ that is a perfect J-abstraction of $q_{S}$ proceeds as follows. First, it computes the query $q_{G}$ that is the UCQ-minimally complete J-abstraction of $q_{S}$. Then, it checks whether $q_{G}$ is a sound abstraction of $q_{S}$ (as discussed above). If the answer is negative, then there exists no UCQ that is a perfect J-abstraction of $q_{S}$. If the answer is positive, then $q_{G}$ is actually a UCQ, and is the perfect J-abstraction of $q_{S}$. Thus the algorithm also solves the computation problem for perfect abstractions in the UCQ language.

** Theorem 6**. [Cima et al. (2019)] Checking whether there exists a query *q* in the class UCQ that is the perfect J-abstraction of $q_{S}$ is decidable. Moreover, there is an algorithm that computes *q*, whenever it exists.

## 6. Monotone abstractions

The notion of monotonicity defines a very natural class of queries that is popular in the field of databases and knowledge representation alike. The intuition behind monotone queries is simple: a query *q* is monotone if, whenever the data we posses increases, the answers for *q* do not decrease. In the literature, however, this notion has been formalized in two distinct ways. In the context of databases, a T-query *q* is monotone if, for every pair of T-databases *D, D*′ such that *D*⊆*D*′, we have *q*^*D*^⊆*q*^*D*^′. Even very simple FOL queries can be shown not to be monotone under this notion. On the other hand, in the context of mathematical logic, the notion of monotonicity comes in a different flavor: a T-query *q* is monotone, if, for every every set of interpretations Σ, Σ′ for T such that Σ⊆Σ′, we have *q*^Σ^⊆*q*^Σ^′. We observe here that, under the semantics of certain answers, FOL queries are monotone in this sense.

To define the notion of monotone queries in the context of a data integration system, we use the notion of monotonicty from logic. A G-query *q* is *monotone in the context of a data integration system* J=〈G,S,M〉 if for every pair *D, D*′ of S-databases, $mod\left(J,D\right)\subseteq mod\left(J,D^{\prime }\right)$ implies $q^{J,D^{\prime }}\subseteq q^{J,D}$. In the following, we use 𝔐^*J*^ to denote the class of monotone queries in the context of J=〈G,S,M〉, and when J is understood, we simply use 𝔐.

This notion of monotonicity is natural yet broad enough to characterize some of the most popular classes of queries. For example, it is trivial to see that queries evaluated under certain answer semantics are monotone. In the light of this consideration, it is natural to ask whether perfect and approximated abstractions in the class of monotone queries always exist for a given class of source queries and whether they can be computed. Moreover, one can show that, whenever an 𝔐-maximally sound (resp., 𝔐-minimally complete) J-abstraction exists, then it is unique. Therefore, from now on, given a source query $q_{S}$, we will talk about the 𝔐-maximally sound (resp., the 𝔐-minimally complete) J-abstraction of $q_{S}$.

In the remainder of this section, we survey recent results on monotone abstractions of UCQs presented in Cima et al. (2022). We introduce a language of monotone queries, called DD^**K**^, with attractive computational properties (Section 6.1). For the case of data integration systems with no axioms in both the global schema and in the source schema, we show that minimally complete and maximally sound monotone abstractions for UCQ source queries always exist, and are expressible in DD^**K**^. From these results, we also derive the decidability of checking whether a perfect monotone abstraction of a given source query exists (Section 6.2).

### 6.1. A language for monotone abstractions

Monotone queries form a natural yet expressive class of queries. Unsurprisingly, perfect and approximated monotone abstractions require a suitably expressive query language. We now introduce one such language and discuss some of its most compelling computational characteristics. The language, called DD^**K**^, is based on disjunctive Datalog, extended with an epistemic operator. We present it in a form specifically tailored for querying data integration systems.

Assume a data integration system J=〈G,M,S〉 and an alphabet of predicate symbols *Int*, called *intensional predicate symbols*, disjoint from the alphabets of G and S. We now consider the case where the logical theories corresponding to both G and S may have a nonempty set of axioms.

A DD^**K**^ query for J includes a set of rules, each one of two possible forms:

- the typical form of disjunctive Datalog, i.e.,

(1) $$\begin{array}{l} b_{1}\wedge \ldots \wedge b_{m}\to i_{1}\vee \ldots \vee i_{n} \end{array}$$

where *b*_1_, …, *b*_*m*_ and *i*_1_, …, *i*_*n*_ are atoms on intensional predicates, and

- a new form specified as follows

(2) $$\begin{array}{l} \text{K}\left(\phi_{1}\left(\bar{x}\right)\vee \ldots \vee \phi_{m}\left(\bar{x}\right)\right)\to \underset{i\in \left\{1..n\right\}}{\vee }\exists \bar{y_{i}}\text{.}\psi_{i}\left(\bar{x},\bar{y_{i}}\right) \end{array}$$

where each ψ_*i*_ is a conjunction of atoms over *Int*, and each ϕ_*i*_ is of the form $\exists \bar{z}\text{.}\gamma \left(\bar{x},\bar{z}\right)\wedge \xi \left(\bar{x}\right)$, with $\gamma \left(\bar{x},\bar{z}\right)$ a conjunction of atoms over G, and $\xi \left(\bar{x}\right)$ a conjunction of inequalities over variables in $\bar{x}$ only.

An *n*-ary DD^**K**^ query *q* for J is a pair q=〈Ans,R〉 where R is a finite set of DD^**K**^ rules, called the definition of *q*, and *Ans* is an *n*-ary intensional predicate in *Int*, called the answer predicate of *q*.

Answers for DD^**K**^ queries are defined based on the notions presented in Calvanese et al. (2007a). An interpretation for *q* is a pair *I* = (*E, f*), where *E* is a set of interpretations for J, and *f* is an interpretation for *Int* with domain *C*. An interpretation *I* = (*E, f*) satisfies a DD^**K**^ rule ρ of *q* (written *I*⊧ρ) if the following conditions hold:

- If ρ is a formula of the form (1), then *I*⊧ρ if *f*⊧ρ, i.e., *f* satisfies the implication in (1).
- If ρ is a formula of the form (2), then *I*⊧ρ if for all tuples $\bar{c}$ of values in *C*, if *I* satisfies the epistemic formula $\text{K}(\wedge_{i}\phi_{i}(\bar{c}))\}$, then there is *j* such that $\exists {ȳ}_{j}\text{.}\psi_{j}\left({\bar{c}}_{j},{ȳ}_{j}\right)$ is true in *f*.

An interpretation *I* for *q* is called a *model* of *q* if all the rules in the definition of *q* are satisfied by *I*. It should be clear that, under this definition of semantics, **K** represents the “knowledge" operator of the modal logic system S5. In other words, the formula **K**α should be read as “α is known (i.e., logically implied) by the system”.

We are ready to define what is the answer $q^{J,D}$ of a DD^**K**^ query q=〈Ans,R〉 with respect to J and the S-database *D*. Specifically, $q^{J,D}=\cap \{\bar{c}\in Ans^{f}|(mod(J,D),f)$ is a model of *q*}.

While a thorough analysis of DD^**K**^ is outside the scope of the present work, we mention some of its most appealing characteristics. Firstly, we observe that DD^**K**^ generalizes UCQs. In particular, every UCQ *q* of *m* disjuncts is equivalent to a DD^**K**^ query with one rule of the form (2) where the disjuncts of *q* are in the scope of **K**. Secondly, every DD^**K**^ query *q* over J is monotone in the context of J. Intuitively, monotonicity follows from a simple form of stratification where certain answers to UCQs (rules of the form (2)) and recursive computations (rules (1)) never mix. In turn, this simple form of stratification guarantees that answering *q* over J boils down to the following: (*i*) computing certain answers for the UCQs in the scope of **K** in the left-hand side of rules of the form (1) in *q*, and (*ii*) computing the answers for the remaining rules (form (2)) over the result of the previous step. Monotonicity follows from the monotonicity of certain answers to UCQs, and from the fact that the rules of the form (2) define a monotone query. These considerations indicate a third appealing characteristic of DD^**K**^. Specifically, the decidability of answering a DD^**K**^ query *q* w.r.t. J and *D* depends exclusively on the decidability of answering UCQs over J, as the following proposition shows.

** Proposition 3**. [(Cima et al., 2022, Proposition 2)] Answering DD^**K**^ queries w.r.t. J and *D* is decidable if and only if computing the certain answers of UCQs w.r.t. J and *D* is decidable.

These results sharply contrast with similar results obtained for plain (non-disjunctive) Datalog. In particular, the undecidability of the latter can be proved even in the case of global schema axioms expressed in very simple Description Logics of the *DL-Lite* family (see, e.g., Levy and Rousset, 1998; Calvanese and Rosati, 2003).

### 6.2. Monotone abstractions via DD^**K**^

We now turn our attention to monotone abstractions expressed in DD^**K**^. We start by observing that, in terms of computational complexity, DD^**K**^ perfectly fits the problem of computing approximated abstractions, as the following proposition shows.

** Proposition 4**. [(Cima et al., 2022, Proposition 3)] There exists a data integration system J with PGAV mapping and a UCQ $q_{S}$ such that answering the 𝔐-maximally sound J-abstraction of $q_{S}$ is coNP-hard in data complexity.

In the remainder of this section, we show that DD^**K**^ is well-suited to express monotone abstractions, both perfect and approximated. In discussing this issue, we go back to our assumption of dealing with data integration systems with no axioms in both the global and the source schema. So, in what follows, we implicitly deal with a data integration system J=〈G,M,S〉, where G and S have no axioms, and a UCQ S-query $q_{S}=q_{1}\cup \ldots \cup q_{n}$, where $q_{i}=\left\{\bar{x}|\exists {ȳ}_{i}.\phi \left(\bar{x},ȳ\right)\right\}$, for *i* = 1, …, *n*.

#### 6.2.1. 𝔐-maximally sound abstractions

In Cima et al. (2022), it is shown that DD^**K**^ can always express 𝔐-maximally sound J-abstractions of UCQs, by illustrating a technique that, given query $q_{S}$, builds a set $R_{J}$ of DD^**K**^ rules whose intensional predicates are the predicates in S, and then uses such rules to construct the 𝔐-maximally sound J-abstractions of $q_{S}$ as a DD^**K**^ query. We do not describe the technique in detail here. Rather, we use an example to give an intuition of the construction.

** Example 8**. Given the following mapping in J:

$\begin{array}{l} m_{1}:\exists y\text{.}s_{1}(x)\wedge s_{2}(x,y)\to g_{1}(x,x)\\ m_{2}:s_{1}(x)\wedge s_{3}(x,y)\to g_{1}(x,y)\\ m_{3}:s_{4}(x)\to \exists y\text{.}g_{1}(x,y) \end{array}$

$R_{J}$ is the following set of DD^**K**^ rules:

$\begin{array}{l} K(g_{1}(x,x))\to (\exists y\text{.}s_{1}(x)\wedge s_{2}(x,y))\vee (s_{1}(x)\wedge s_{3}(x,x))\\ K(g_{1}(x,y)\wedge x\neq y)\to s_{1}(x)\wedge s_{3}(x,y)\\ K(\exists y\text{.}g_{1}(x,y))\to s_{4}(x)\vee (\exists y\text{.}s_{1}(x)\wedge s_{3}(x,y))\vee \\ \;(\exists y\text{.}s_{1}(x)\wedge s_{2}(x,y)) \end{array}$

Intuitively, the rules of $R_{J}$ specify, for the various facts over G that are certain, i.e., that are *known* to hold, the queries over the sources that generate them. For example, the first rule of $R_{J}$ specifies that, if a constant is known to satisfy *g*_1_(*x, x*), then this knowledge derives either from the answers to the source query {*x*|∃*y*.*s*_1_(*x*)∧*s*_2_(*x, y*)} or from the answers to the source query {*x*|*s*_1_(*x*)∧*s*_3_(*x, x*)}. As another example, the second rule of $R_{J}$ specifies that the pairs of distinct constants *x, y* known to satisfy *g*_1_(*x, y*) derive from the query {*x, y*|*s*_1_(*x*)∧*s*_3_(*x, y*)}. It can be shown that this is crucial for ensuring that the abstraction of queries involving the join of *s*_1_ and *s*_3_, which is based on the certain answers of *g*_1_, do not include data deriving from source queries whose abstraction is based on the certain answers of the projection of *g*_1_. Finally, the third rule of $R_{J}$ takes care of those constants *x* known to satisfy *g*_1_(*x, y*), for some, not necessarily known, *y*. Such constants may derive from each of source queries above.

Using the notion of $R_{J}$, we can immediately obtain the 𝔐-maximally sound J-abstraction of $q_{S}$, by adding to $R_{J}$ the set A constituted by one rule of the form $\phi_{i}\left(\bar{x},ȳ\right)\to Ans\left(\bar{x}\right)$ for each disjunct $q_{i}=\left\{\bar{x}|\exists {ȳ}_{i}.\phi \left(\bar{x},ȳ\right)\right\}$ in $q_{S}$.

** Proposition 5**. [(Cima et al., 2022, Theorem 2)] The DD^**K**^ query $\langle Ans,R_{J}\cup A\rangle$ is the 𝔐-maximally sound J-abstraction of $q_{S}$.

In the light of Proposition 5 and from the existence of an algorithm to compute $R_{J}\cup A$, we obtain the following.

** Theorem 7**. [(Cima et al., 2022, Theorem 2)] The 𝔐-maximally sound J-abstraction of $q_{S}$ always exists, is computable, and can be expressed in DD^**K**^.

#### 6.2.2. 𝔐-minimally complete abstractions

We show that DD^**K**^ can always express 𝔐-minimally complete J-abstractions of UCQs.

Let us first introduce a useful notion. Given a CQ $q=\left\{\bar{x}|\exists ȳ\text{.}\phi \left(\bar{x},ȳ\right)\right\}$, Saturate(*q*) denotes the UCQ with inequalities obtained as follows. For each possible unifier μ on the variables in $\bar{x}\cup ȳ$ such that $\mu \left(x\right)\in \bar{x}$ for each $x\in \bar{x}$, Saturate(*q*) contains a query obtained from μ(*q*) by adding an inequality atom (*t*_1_≠*t*_2_) for each pair of distinct variables *t*_1_, *t*_2_ occurring in μ(*q*). For a UCQ *Q*, we denote by Saturate(*Q*) the UCQ with inequalities consisting of the union of Saturate(*q*), for each disjunct *q* of *Q*. It is easy to see that Saturate(*Q*) is equivalent to *Q*, for every UCQ *Q*.

Consider a disjunct *q*_*h*_ in in $Saturate\left(q_{S}\right)$. Clearly, *q*_*h*_ is a CQ with inequalities of the form $q_{h}=\left\{\bar{x}|\exists ȳ\text{.}\phi \left(\bar{x},ȳ\right)\wedge \chi \left(\bar{x},ȳ\right)\right\}$, where $\chi \left(\bar{x},ȳ\right)$ are inequality atoms. Let $M\left(q_{h}\right)$ denote the result of chasing the set of relational atoms occurring in *q*_*h*_ with M. Let ρ_*q*_*h*__ denote the DD^**K**^ rule $\text{K}\left(M\left(q_{h}\right)\wedge ⊤\left(\bar{x}\right)\wedge \chi \left(\bar{x},ȳ\right)\right)\to Ans\left(\bar{x}\right)$. Finally, let *q*_*c*_ denote the DD^**K**^ query consisting of all the rules ρ_*q*_*h*__ for the various *q*_*h*_ in $Saturate\left(q_{S}\right)$ and with answer predicate *Ans*. We can now prove the following.

** Proposition 6**. [(Cima et al., 2022, Theorem 1)] *q*_*c*_ is the 𝔐-minimally complete J-abstraction of $q_{S}$.

The following statement is a straightforward consequence of Proposition 6.

** Theorem 8**. [(Cima et al., 2022, Theorem 1)] The 𝔐-minimally complete J-abstraction of $q_{S}$ always exists, is computable, and can be expressed in DD^**K**^.

#### 6.2.3. Perfect monotone abstractions

From the results presented above, we can derive an algorithm for checking whether there exists a query in 𝔐 that is the perfect J-abstraction of $q_{S}$. In particular, observe that if the perfect J-abstraction of $q_{S}$ can be expressed as a query in 𝔐, then it is J-equivalent to the 𝔐-minimally complete J-abstraction of $q_{S}$. Then, from Proposition 6 we know that, in order to check whether there exists a query in 𝔐 that is the perfect J-abstraction of $q_{S}$, we have to check whether $q_{S}$ is equivalent to *q*_*c*_ modulo J.

To this end, we observe the following. There exists a UCQ with inequalities S-query *q*_*min*_ such that $q_{min}^{D}=q_{c}^{J,D}$, for every S-database *D*. Moreover, *q*_*min*_ is computable. These two properties result from J being a GLAV data integration system with no source and global schema axioms, and from the specific form of *q*_*c*_. Therefore, in order to check whether there exists a query in 𝔐 that is the perfect J-abstraction of $q_{S}$, we just need to check whether $q_{min}⊑q_{S}$. The next claim follows from these considerations.

** Theorem 9**. [(Cima et al., 2022, Theorem 3)] Checking whether there exists a query *q* in the class 𝔐 that is the perfect J-abstraction of $q_{S}$ is decidable. Moreover, there is an algorithm that computes *q*, whenever it exists.

## 7. Non-monotone abstractions

So far, we have limited our analysis of the abstraction reasoning task by focusing on monotone query languages in the context of data integration systems. There exist, however, very simple scenarios in which the perfect abstraction can only be expressed by means of a non-monotone query.

** Example 9**. Let J=〈G,S,M〉 be such that the global schema G has the predicates {*A*/1, *B*/1, *C*/1}, the source schema S has the predicates {*s*_1_/1, *s*_2_/1}, and $M=\left\{m_{1},m_{2},m_{3},m_{4}\right\}$, where:

$\begin{array}{l} m_{1}:s_{1}(x)\to A(x)\\ m_{2}:s_{2}(x)\to A(x)\\ m_{3}:s_{2}(x)\to B(x)\\ m_{4}:s_{1}(x)\wedge s_{2}(x)\to C(x) \end{array}$

Consider the query $q_{S}=\left\{x|s_{1}\left(x\right)\right\}$. One can verify that the perfect J-abstraction of $q_{S}$ is the non-monotone query $q_{G}$ such that, given an S-database *D*, returns those *x* for which either (*A*(*x*)∧¬*B*(*x*)) or *C*(*x*) is known to be true, i.e. holds in every G-database *B* such that B∈mod(J,D).

Motivated by the above example, in this section we summarize the most salient aspects of the results in Cima et al. (2020), which investigates the problem of finding perfect (resp. minimally complete, maximally sound) abstractions expressed in the query language *EQL-Lite*(UCQ).^4^ For instance, refer to Example 9. The perfect J-abstraction of $q_{S}$ written there in natural language can be formulated through the *EQL-Lite*(UCQ) query $q_{G}=\left\{\left(x\right)|\text{K}\left(A\left(x\right)\wedge \neg B\left(x\right)\right)\vee \text{K}\left(C\left(x\right)\right)\right\}$. As in the case of the UCQ and the 𝔐 classes, it can be shown that if an *EQL-Lite*(UCQ)-maximally sound (resp., *EQL-Lite*(UCQ)-minimally complete) J-abstraction of $q_{S}$ exists, then it is unique up to J-equivalence. Thus, in what follows, we will simply talk about *the* *EQL-Lite*(UCQ)-maximally sound (resp., *EQL-Lite*(UCQ)-minimally complete) J-abstraction of $q_{S}$.

A natural question that arises is whether “best” abstractions in the *EQL-Lite*(UCQ) query language always exist. Unfortunately, the following theorem shows that this is not the case for both *EQL-Lite*(UCQ)-minimally complete abstractions and *EQL-Lite*(UCQ)-maximally sound abstractions.

** Theorem 10**. [(Cima et al., 2020, Theorems 1 and 2)] Both the *EQL-Lite*(UCQ)-minimally complete J-abstractions of $q_{S}$ and the *EQL-Lite*(UCQ)-maximally sound J-abstractions of $q_{S}$ may not exist.

Due to the above negative result, which holds already for CQJFE queries $q_{S}$ and data integrations systems with PGAV mappings, we now explore two alternative restricted scenarios. The former weakens the target query language for expressing abstractions by considering a fragment of *EQL-Lite*(UCQ), whereas the latter weakens the mapping language by considering a special case of GLAV. In both the restricted scenarios, we assume that source queries are CQs rather than UCQs.

### 7.1. A restricted non-monotone query language

We now consider the problem of finding abstractions expressed in *EQL-Lite*^−^(UCQ), which corresponds to the fragment of *EQL-Lite*(UCQ) where both *nested* negation and union operators are disallowed. More formally, an *EQL-Lite*^−^(UCQ) query *q* is an expression of the form $q=\left\{\bar{x}|\varphi \left(\vec{x}\right)\right\}$ where $\varphi \left(\vec{x}\right)$ is an *EQL* formula built according to the following syntax:


$$\begin{array}{l} \varphi ::=\;\text{K}\varrho |\exists y.\varphi |\varphi_{1}\wedge \varphi_{2}|\;\neg \delta \\ \delta ::=\;\text{K}\varrho |\exists y.\delta \end{array}$$

with ϱ being a disjunction of conjunction of atoms over G possibly involving existentially quantified variables. For instance, the *EQL-Lite*(UCQ) query $q_{G}$ illustrated above, which corresponds to the perfect J-abstraction of $q_{S}$ in Example 9, is not an *EQL-Lite*^−^(UCQ) query.

On the negative side, even in this scenario, maximally sound abstractions are not guaranteed to exist, and this holds already for CQJFE queries $q_{S}$ and data integrations systems with PGAV mappings.

** Theorem 11**. [(Cima et al., 2020, Theorem 2)] The *EQL-Lite*^−^(UCQ)-maximally sound J-abstractions of $q_{S}$ may not exist.

On the positive side, we now provide an algorithm for computing *EQL-Lite*^−^(UCQ)-minimally complete J-abstractions of CQs $q_{S}$. The algorithm is similar to the one for the UCQ case (cf. Section 5), expect that all the atoms obtained when applying the mapping to the given CQ occur inside the scope of the epistemic operator **K**, binding also the existential variables coming from the input query. More precisely, given a data integration system J=〈G,S,M〉 and a CQ $q_{S}=\left\{\bar{x}|\exists ȳ\text{.}\phi \left(\bar{x},ȳ\right)\right\}$, the algorithm returns the *EQL-Lite*^−^(UCQ) query $q_{G}=\left\{\bar{x}|\exists \bar{Y}\text{.}\text{K}\left(\exists \bar{z}\text{.}M\left(q_{S}\right)\wedge ⊤\left(\bar{x}\right)\right)\right\}$, where $\bar{Y}\subseteq ȳ$ are the existential variables of $q_{S}$ occurring in $M\left(q_{S}\right)$, while $\bar{z}$ are the fresh existential variables introduced when applying M to $q_{S}$. To see the difference with the UCQ case, recall Example 1 in the introduction and the CQ $q_{S}^{2}$ therein. While $q_{G}^{2}$ is the UCQ-minimally complete J-abstraction of $q_{S}^{2}$, the *EQL-Lite*^−^(UCQ) query {*x*∣∃*y*.**K**(*g*_1_(*x, y*))} returned by the above algorithm is a better complete approximation than $q_{G}^{2}$, and is in fact the perfect J-abstraction of $q_{S}^{2}$.

** Theorem 12**. [(Cima et al., 2020, Theorem 5)] The *EQL-Lite*^−^(UCQ)-minimally complete J-abstraction of a CQ $q_{S}$ always exists and is computable.

We further notice that the above algorithm returns queries that are monotone and that are expressible in DD^**K**^, thus proving that, without disjunction, the limited form of negation allowed in *EQL-Lite*^−^(UCQ) does not give more expressive power in finding minimally complete (and therefore also perfect) abstractions of CQs. On the contrary, it can be shown that inequalities give more expressive power in finding abstractions. In particular, there exist 𝔐-minimally complete J-abstractions of CQs that cannot be expressed in *EQL-Lite*^−^(UCQ), whereas, as shown in the previous section, they can be expressed in DD^**K**^.

Given a query $q_{G}$ as returned by the above algorithm, it is always possible to compute a UCQ *q*_*u*_ such that $q_{u}^{D}=q_{G}^{J,D}$ for every S-database *D*. Thus, following the same line of reasoning as the one at the end of the previous section, in this scenario we can solve the computation problem for perfect abstractions of CQs.

** Theorem 13**. [Cima et al. (2020)] Checking whether there exists a query *q* in *EQL-Lite*^−^(UCQ) that is the perfect J-abstraction of a CQ $q_{S}$ is decidable. Moreover, if it exists, then *q* is a monotone query and there is an algorithm that computes it.

### 7.2. One-to-one mapping

We now examine the problem of finding abstractions in the presence of data integration systems J=〈G,S,M〉 such that M is a *one-to-one mapping*. A one-to-one mapping is a special case of GLAV, constituted by a set of assertions of the form $\exists ȳ\text{.}s\left(\bar{x},ȳ\right)\to \exists \bar{z}\text{.}g\left(\bar{x},ȳ\right)$, where $s\left(\bar{x},ȳ\right)$ and $g\left(\bar{x},ȳ\right)$ are single atoms without constants or repeated variables.

The first result is that the algorithm previously presented for computing *EQL-Lite*^−^(UCQ)-minimally complete abstractions of CQs can be also used for computing *EQL-Lite*(UCQ)-minimally complete abstractions of CQs for data integration systems J with PGAV mapping.

** Theorem 14**. [(Cima et al., 2020, Theorem 3)] Under one-to-one mappings, the *EQL-Lite*(UCQ)-minimally complete J-abstraction of a CQ $q_{S}$ always exists, is computable, and is a monotone query.

Thus, using exactly the same considerations done for the case of *EQL-Lite*^−^(UCQ), we can solve the computation problem for perfect abstractions in *EQL-Lite*(UCQ) of CQs under one-to-one mappings.

** Theorem 15**. [Cima et al. (2020)] Under one-to-one mappings, checking whether there exists a query *q* in *EQL-Lite*(UCQ) that is the perfect J-abstraction of a CQ $q_{S}$ is decidable. Moreover, if it exists, then *q* is a monotone query and there is an algorithm that computes it.

We now turn to the sound case under one-to-one mappings. Specifically, in this scenario, while the existence of *EQL-Lite*(UCQ)-maximally sound J-abstractions of CQs is still an open problem, we present an algorithm for computing *EQL-Lite*(UCQ)-maximally sound J-abstractions of CQJFEs $q_{S}$. Roughly speaking, given a data integration system J=〈G,S,M〉 with M a one-to-one mapping and a CQJFE $q_{S}$, as a first step the algorithm computes the *EQL-Lite*(UCQ)-minimally complete $q_{G}$ of $q_{S}$ and its UCQ reformulation *q*_*u*_ such that $q_{u}^{D}=q_{G}^{J,D}$ for each S-database *D*. Then, for each CQ *q*′ which is a disjunct of *q*_*u*_ such that $q^{\prime }⋢q_{S}$, the algorithm adds in conjunction to the body of $q_{G}$ the negation of the body of the *EQL-Lite*(UCQ)-minimally complete of *q*′. Informally, this last step prevents $q_{G}$ to return answers that are not answers of $q_{S}$, guaranteeing soundness of the output query. For instance, recall Example 1, and let $J^{\prime }=\langle G,S,M^{\prime }\rangle$ be the data integration system with $M^{\prime }=\left\{m_{1},m_{2},m_{3}\right\}$ a one-to-one mapping. The query returned by the algorithm is the *EQL-Lite*^−^(UCQ) query {*x*∣**K**(*A*(*x*))∧¬**K**(*B*(*x*))}, which is the *EQL-Lite*(UCQ)-maximally sound $J^{\prime }$ abstraction of $q_{S}$.

** Theorem 16**. [(Cima et al., 2020, Theorem 4)] Under one-to-one mappings, the *EQL-Lite*(UCQ)-maximally sound J-abstraction of a CQJFE $q_{S}$ always exists and is computable.

We conclude this section with the following observation. The algorithms sketched above for computing “best” abstractions always return an *EQL-Lite*^−^(UCQ) query. This directly implies that, under one-to-one mappings, the query languages *EQL-Lite*(UCQ) and *EQL-Lite*^−^(UCQ) have the same expressive power in finding all three kinds of abstractions (perfect, minimally complete, and maximally sound).

## 8. Open problems

We have provided an overview of data abstraction, and we have illustrated some results obtained in recent years on computing abstractions. We conclude the paper by discussing a set of issues related to abstractions that deserve more investigation.

### 8.1. Data quality

While data quality is one the main issues proposed in Data-centric AI, there is no general and well-established methodology for leveraging data quality for improving Machine Learning methods.

As pointed out in Chen et al. (2021), poor data quality has a direct impact on the performance of the machine learning system that is built on the data. It is therefore important to devise techniques for validating the quality of both training and testing datasets. Recent work in this direction shows a strong correlation between the quality of the datasets and the performance of the machine learning system, and demonstrates that a rigorous evaluation of data quality is necessary for guiding the quality improvement of machine learning. We believe that formal methods like data abstraction can provide some contributions toward this goal. For example, by helping in making the semantics of training data explicit, abstraction can provide support for recognizing biases or other problems in the data used to train a Machine Learning Model. Making concrete steps in this direction is a stimulating research challenge.

### 8.2. Languages for abstractions

A crucial issue related to abstraction is to compute perfect and approximated abstractions within specific classes of queries. For the fundamental class UCQ, the decidability of checking whether there exists a UCQ-maximally sound abstraction of a UCQ source query is still open. More generally, there are many interesting classes of queries that can be used to express abstractions, and for which it would be interesting to compute perfect, or approximated abstractions. For example, in the case of graph databases as virtual views, relevant classes of queries for abstractions include regular path queries, or two-way conjunctive regular path queries.

### 8.3. Abstraction and monotonicity

In this paper we have discussed the use of DD^**K**^ to express monotone abstractions of source queries in the class UCQ. It would be interesting to investigate which is the minimal expressive power needed for capturing perfect and approximated monotone abstractions of source queries. Also, it is not difficult to see that there are queries for which the perfect abstraction is non-monotone. Although first results on non-monotone abstractions have appeared in Cima et al. (2020), the issue of checking the existence of and computing non-monotone abstractions is largely unexplored.

### 8.4. Expressive source queries

The majority of work on abstraction so far focused on source queries in the class UCQ. It would be interesting to address the problem of computing perfect and approximated abstractions of source queries expressed in more expressive languages such as Datalog. More expressive mapping languages (e.g., UCQ with inequalities in the GLAV type of mapping) also deserve attention.

### 8.5. Axioms

The computation of abstractions in the presence of axioms in the global schema or in the source schema is another interesting problem to study. First results in this direction appeared in Cima (2017), Lutz et al. (2018), and Cima et al. (2019), but the topic requires a more thorough analysis.

### 8.6. Reverse engineering

Abstraction has also interesting connections with the reverse-engineering problem (Barceló and Romero, 2017). When casted in data integration, given a source database *D* and set *P* of tuples, this problem aims at finding a global schema query *q* that captures *P*, i.e., such that the answers of *q* with respect to *D* captures the tuples in *P*. Despite the intuitive connection, a detailed analysis of the relationship between the two problems is missing.

### 8.7. User requirements

Finally, crucial aspects of abstractions, such as succinctness and clarity, have not been considered in this paper. More generally, issues related to the adequacy of the formulation of abstractions with respect to user requirements deserve greater attention.

## Author contributions

All authors listed have made a substantial, direct, and intellectual contribution to the work and approved it for publication.

## Footnotes

^1^In principle, we could also consider databases that are infinite structures.

^2^When we refer to UCQ views, we in fact assume that view definitions are UCQs without *repeated variables* in the target list. We refer to Afrati and Chirkova (2019) for the complications that can arise when this assumption is removed.

^3^This is not the case when view definitions are expressed as *regular path queries* rather than UCQs (Calvanese et al., 2002).

^4^Actually, we consider the slightly restricted version of *EQL-Lite*(UCQ) which does not allow the use of (in)equalities.
